# Selective killing of human immunodeficiency virus infected cells by non-nucleoside reverse transcriptase inhibitor-induced activation of HIV protease

**DOI:** 10.1186/1742-4690-7-89

**Published:** 2010-10-15

**Authors:** Dirk Jochmans, Maria Anders, Inge Keuleers, Liesbeth Smeulders, Hans-Georg Kräusslich, Günter Kraus, Barbara Müller

**Affiliations:** 1 Tibotec-Virco BVBA, Mechelen, Belgium; 2 Department of Infectious Diseases, Virology, University of Heidelberg, Germany; 3 Rega Institute for Medical Research, Katholieke Universiteit Leuven, Minderbroedersstraat 10, B-3000 Leuven, Belgium

## Abstract

**Background:**

Current antiretroviral therapy against human immunodeficiency virus (HIV-1) reduces viral load and thereby prevents viral spread, but it cannot eradicate proviral genomes from infected cells. Cells in immunological sanctuaries as well as cells producing low levels of virus apparently contribute to a reservoir that maintains HIV persistence in the presence of highly active antiretroviral therapy. Thus, accelerated elimination of virus producing cells may represent a complementary strategy to control HIV infection. Here we sought to exploit HIV protease (PR) related cytotoxicity in order to develop a strategy for drug induced killing of HIV producing cells. PR processes the viral Gag and Gag-Pol polyproteins during virus maturation, but is also implicated in killing of virus producing cells through off-target cleavage of host proteins. It has been observed previously that micromolar concentrations of certain non-nucleoside reverse transcriptase inhibitors (NNRTIs) can stimulate intracellular PR activity, presumably by enhancing Gag-Pol dimerization.

**Results:**

Using a newly developed cell-based assay we compared the degree of PR activation displayed by various NNRTIs. We identified inhibitors showing higher potency with respect to PR activation than previously described for NNRTIs, with the most potent compounds resulting in ~2-fold increase of the Gag processing signal at 250 nM. The degree of enhancement of intracellular Gag processing correlated with the compound's ability to enhance RT dimerization in a mammalian two-hybrid assay. Compounds were analyzed for their potential to mediate specific killing of chronically infected MT-4 cells. Levels of cytotoxicity on HIV infected cells determined for the different NNRTIs corresponded to the relative degree of drug induced intracellular PR activation, with CC_50 _values ranging from ~0.3 μM to above the tested concentration range (10 μM). Specific cytotoxicity was reverted by addition of PR inhibitors. Two of the most active compounds, VRX-480773 and GW-678248, were also tested in primary human cells and mediated cytotoxicity on HIV-1 infected peripheral blood mononuclear cells.

**Conclusion:**

These data present proof of concept for targeted drug induced elimination of HIV producing cells. While NNRTIs themselves may not be sufficiently potent for therapeutic application, the results provide a basis for the development of drugs exploiting this mechanism of action.

## Background

Current highly active antiretroviral therapy (HAART), involving combination treatment with three or more antiviral drugs, allows the efficient control of human immunodeficiency virus (HIV) replication. Under optimal conditions, suppression of plasma viral load below the detection limit of standard diagnostic assays (50 RNA copies/ml) can be achieved for prolonged periods of time [1]. However, persistent viremia at very low levels is detected even in these cases using highly sensitive methods [2-4], and treatment interruption, even after years of successful therapy, results in viral rebound [5-8]. Targeted eradication of latently infected cells and of virus producing cellular reservoirs appears to be essential to cure HIV infection, which represents the ultimate goal of antiretroviral therapy.

HIV has evolved mechanisms to influence the balance of death and survival of the host cell in order to promote efficient virus replication [9]. By directly and indirectly destroying cells of the immune system the virus undermines host defense mechanisms. On the other hand, activation and temporary survival of infected immune cells is also essential for productive virus replication. Tipping this delicate balance by drug induced enhancement of HIV mediated cytotoxicity could potentially be exploited as a means for rapid elimination of infected cells. To explore this strategy we focused on the viral protease (PR). While several other HIV encoded proteins, in particular Vpr, Tat, Nef and Vpu, have been reported to play complex roles in cell activation and cell destruction, mainly through induction or inhibition of apoptosis [9], the intricate processes mediated by these accessory proteins are not restricted to the infected cell itself, but can exert bystander effects on non infected cells. In contrast, a more direct role in killing of the infected cell has been suggested for HIV PR. Overexpression of PR in various systems or premature activation of PR in virus producing cells, respectively, has been shown to result in cell death, presumably by off-target cleavage of cellular proteins [10-13]. PR is an aspartic protease expressed as part of the viral Gag-Pol polyprotein precursor. It is encoded in the viral genome as an enzymatically inactive monomer, whose dimerization is required for formation of the active site. Although the mechanism of HIV PR activation in the course of the viral replication cycle is currently not fully understood, it is believed that PR dimer formation through dimerization of the Gag-Pol precursor does play a role in this process.

PR is essential for proteolytic processing of the viral Gag and Gag-Pol precursor proteins into their functional subunits. This process occurs concomitant with or shortly after particle release [14] and results in morphological maturation of the virion into its infectious form. Enhanced or premature processing of precursor proteins prevents their assembly into an immature viral particle [12,15-17]; the temporal regulation of proteolytic maturation is thus crucial for HIV replication. This involves an ordered series of cleavage events at distinct processing sites within the Gag and Gag-Pol polyproteins, which differ in amino acid sequence and susceptibility to PR processing [18-20]. Due to the relaxed substrate specificity of HIV PR the enzyme does not exclusively recognize the viral polyproteins, but is also able to catalyze the cleavage of a number of host cell proteins including actin [21], vimentin [22], Bcl-2 [13], poly A binding protein [23], eIF4G [24] and procaspase 8 [25]. Proteolysis of host cell factors offers an explanation for the cytotoxic effect of the HIV PR protein, which has been observed in various cell types upon overexpression of PR [10,11] or upon premature activation of PR through artificial joining of two monomeric PR domains [16]. The relevance of PR cleavage of particular host cell proteins for HIV infection is currently unclear. However, it has been reported that PR mediated cleavage of procaspase 8 can be responsible for specific killing of HIV infected T-cells [26].

Based on these data, augmenting intracellular PR activity, e.g. by increasing Gag-Pol dimer formation, should result in enhancement of HIV mediated cytotoxicity and thus selective killing of infected cells. To test this hypothesis we made use of the fact that drug induced enhancement of HIV-1 PR activity has already been described for one class of currently used antiretroviral drugs, namely non-nucleoside inhibitors of HIV-1 reverse transcriptase (NNRTIs) [27]. NNRTIs are an integral part of modern HAART regimens [28]. They bind to a hydrophobic pocket within the palm subdomain of HIV-1 reverse transcriptase (RT) and inhibit its DNA polymerase activity in an allosteric manner. Like PR, RT is encoded as part of the Gag-Pol polyprotein and needs to dimerize in order to display enzymatic activity [29,30]. The mature enzyme consists of p66, comprising the polymerase and RNase H active sites, and its 51 kDa subfragment lacking the C-terminal RNase H domain. Mutational analyses indicate that RT residues close to the NNRTI binding region are important for RT dimer stability [31]. Using yeast two-hybrid assays or biochemical methods, respectively, it has been shown that binding of some NNRTI compounds can shift the monomer-dimer equilibrium of p66 containing proteins towards the dimeric form [27,32-35]. This correlates with the observation that these NNRTIs lead to an increase in intracellular Gag-Pol and Gag processing by PR, suggesting that this is due to an enhancement of Gag-Pol dimerization. Since premature Gag proteolysis results in reduced or abolished particle formation [12,15-17], it has been proposed that this mechanism could be an alternative principle of HIV inhibition by NNRTIs. However, NNRTIs induce only partial inhibition of virion release and the drug concentrations required are several orders of magnitude higher than those resulting in efficient inhibition of RT activity [27].

Here, we investigate whether drug mediated PR activation can be exploited to induce specific killing of HIV infected cells. Applying a newly developed cell based assay system we compared the efficacy of various NNRTIs with respect to the enhancement of intracellular Gag and Gag-Pol processing. Using the two most potent compounds tested, we showed specific killing of HIV producing T-cell lines or primary T-cells, which was dependent on PR activity. The results obtained provided proof of principle validation of this strategy and can serve as a basis to search for more potent small molecule enhancers of Gag-Pol dimer formation.

## Results

### Development of a cell based assay to measure intracellular Gag processing

**Figure 1:**
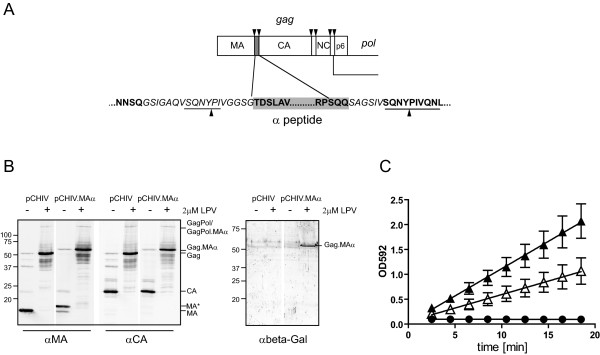
**Construction and characterization of an HIV derivative carrying the β-Gal alpha peptide**. **(A) **The coding sequence for amino acids 1-51 of β-Gal (gray box) was inserted into the *gag *open reading frame of plasmid pCHIV. Amino acids displayed in bold represent authentic sequences from HIV Gag or β-Gal, respectively, while introduced linker sequences are displayed in italics. Arrowheads indicate cleavage sites for HIV PR. **(B) **Immunoblot analysis of HIV.MAα particles. 293T cells transfected with the indicated constructs were grown in the absence (-) or presence (+) of 2 μM LPV. At 44 h post transfection, particles were purified by ultracentrifugation and analyzed by immunoblotting using the indicated antisera. Molecular mass standards (in kDa) are shown on the left, specific protein products are identified on the right. **(C) **β-Gal activity in lysates of transfected 293T cells dependent on HIV PR activity. Cell lysates from untransfected 293T cells (filled circles), or from 293T cells transfected with a mixture of pCMVω and pCHIV.MAα and incubated in the presence of DMSO (filled triangles) or 2 μM LPV (open triangles, respectively, were prepared at 48 h post transfection and β-Gal activity was determined *in vitro *through cleavage of the colorimetric substrate CPRG by measuring changes in OD592 over time. The graph shows mean values and standard deviations from five independent experiments. Relative rates of CPRG cleavage were determined by linear regression, yielding an average value of 0.109 min^-1 ^for the DMSO controls and 0.054 min^-1 ^for the LPV treated samples, respectively

In previous studies, high concentrations of NNRTI (5 μM) were required to observe NNRTI mediated activation of intracellular HIV PR activity [27]. Furthermore, not all NNRTI compounds tested were found to be equally active: while 5 μM of efavirenz (EFV), etravirine (ETV) or TMC-120, respectively, have been reported to resulted in a similar enhancement of processing activity, nevirapine (NVP) or delavirdine (DLV) did not stimulate Gag or Gag-Pol processing under the conditions used [27]. Hence, before testing the potential of NNRTI compounds for HIV infected cell killing we wanted to identify the most potent compound available. Towards this end, we developed a biochemical assay for gel independent quantitation of intracellular Gag processing by HIV PR in the context of a virus producing cell. We had previously shown that additional protein domains, consisting of small epitope tags or even the 27 kDa green fluorescent protein (EGFP), can be inserted between the MA and CA domains of the Gag and Gag-Pol polyproteins without affecting polyprotein production or processing by HIV PR [36]. Based on this, we designed a HIV reporter construct which contained a small N-terminal fragment ('alpha peptide') of *Escherichia coli *beta-galactosidase (β-Gal), flanked by two HIV PR recognition sites, between the MA and CA coding sequences of Gag (Figure 1A). Co-expression of the alpha peptide together with the larger C-terminal portion ('omega subunit') of β-Gal results in restoration of enzymatically active tetrameric β-Gal through the intracellular association of the two enzymatically inactive fragments. This so called alpha complementation principle can be exploited for use in mammalian cells [37,38] and has been employed for the establishment of various cell based biochemical assay systems [39]. We reasoned that embedding of the small alpha peptide within the multi-domain polyproteins Gag or Gag-Pol, respectively, should impair its productive association with the omega subunit, while proteolytic release of the alpha peptide from the polyprotein by PR would allow the formation of enzymatically active β-Gal. This should allow us to monitor intracellular Gag and Gag-Pol processing through increased β-Gal activity.

The reporter virus was generated by inserting the coding sequence for amino acids 1-51 of β-Gal (defined as the minimal complementary peptide in [40]) at the 3' end of the MA coding region of proviral plasmid pNLC4-3, resulting in plasmid pNLC4-3.MAα. In order to allow specific release of the alpha peptide from this modified polyprotein by HIV-1 PR, the peptide sequence was flanked by short linker sequences and two SQNYPIV motifs (Figure 1A, underlined) based on the PR recognition site between HIV-1 MA and CA. Processing by HIV PR at these sites would yield free alpha peptide flanked by short linker sequences, the authentic CA protein, as well as MA extended by a 9 amino acid linker insertion (SQGSIGAQV) at its C-terminus (Figure 1A). Construct pCHIV.MAα was based on the non-infectious pNL4-3 derivative pCHIV, which expresses all viral proteins except Nef, but cannot replicate due to the lack of both viral long terminal repeat regions [41]. Particles were prepared from the supernatant of 293T cells transfected with pCHIV.MAα in the presence and absence of PR inhibitor (PI) and analyzed for the presence of the modified Gagα protein by immunoblot. Gag containing particles were released from pCHIV.MAα transfected cells with comparable efficiency as wild type pCHIV derived particles and processing was blocked by the specific PI lopinavir (LPV) (Figure 1B). A slightly reduced electrophoretic mobility of the Gag precursor in the pCHIV.MAα transfected cells, as well as the reactivity of the polyprotein with antiserum against β-Gal indicated the presence of the alpha peptide. Processing products of the modified Gag precursor were identical to those of wild-type Gag, with the exception of a slightly slower migrating form of MA (MA*), presumably representing mature MA extended by the 9 amino acid linker sequence preceding the cleavage site between MA and the alpha peptide retained only on a part of the MA molecules. The free alpha peptide was not detectable by immunoblot analyses. When the alpha peptide was inserted in the context of the replication competent provirus HIV-1_NL4-3_, no impairment of virus replication was observed compared to wild-type HIV-1 (see Additional file 1 for infectivity data).

Having established that the MAα modification did not affect the properties of the virus in tissue culture, we tested whether Gag processing could be measured via proteolytic release of the alpha peptide and subsequent reconstitution of β-Gal activity by association with the omega fragment. 293T cells were co-transfected with pCHIV.MAα and pCMVω, which encodes an inactive fragment of β-Gal lacking amino acids 11-41 under the control of the CMV promoter. Reconstituted β-Gal activity in cell lysates was measured by cleavage of the chromogenic substrate CPRG [42] as described in Methods. As shown in Figure 1C, lysates from untransfected cells (filled circles) lacked detectable activity, while lysates from cells co-transfected with pCMVω and pCHIV.MAα (filled triangles) displayed β-Gal activity. To test whether the enzymatic activity measured reflected HIV-1 PR mediated release of the alpha peptide from the Gagα precursor, transfected cells were incubated in the presence of 2 μM LPV, which nearly completely blocked Gagα processing as determined by immunoblot. This treatment reduced, but did not abolish, β-Gal activity in the cell lysates (Figure 1C, open triangles); a similar level of residual activity was also observed when PR activity and Gag processing was completely blocked by a D25A mutation in the PR active site (not shown), suggesting that some complementation by the alpha peptide can occur when the peptide is inserted within an extended and flexible region of the Gag-Pol polyprotein. Nevertheless, PR inactivation resulted in significantly reduced relative β-Gal activities of cell lysates as compared to the DMSO control (p = 0.0006 for the data shown in Figure 1C, analyzed by a paired two-tailed t-test).

### Effect of different NNRTIs on intracellular Gag processing

**Figure 2:**
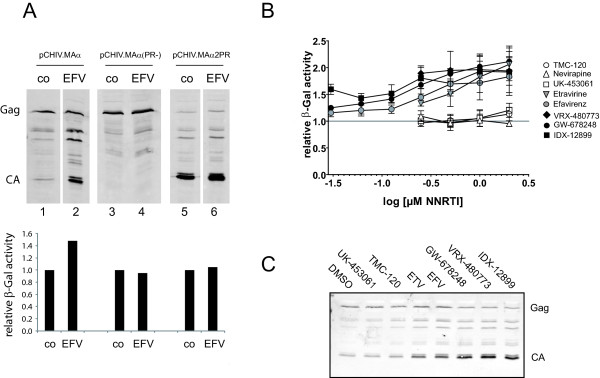
**Effect of NNRTIs on alpha complementation and intracellular Gag processing efficiency**. **(A)** 293T cells transfected with a mixture of pCMVω and pCHIV.MAα (lanes 1-2), pCHIV.MAα(PR-) (lanes 3-4), or pCHIV.MAα2PR (lanes 5-6), respectively, were incubated in the presence of DMSO (lanes 1, 3 and 5), or 5 μM EFV (lanes 2, 4 and 6). At 44 h post transfection, cell lysates were harvested and analyzed by immunoblot using antiserum raised against HIV CA (top), as well as for relative β-Gal activity (bottom). CPRG cleavage rates determined as described in materials and methods were normalized to the value obtained for the respective solvent control. **(B) **293T cells transfected with a mixture of pCHIV.MAα and pCMVω were grown in the presence of DMSO or 0.25 to 10 μM of the indicated NNRTI, respectively. At 44-48 h post transfection, cell lysates were harvested and analyzed for β-Gal activity. The graph shows mean CPRG cleavage rates and standard deviations from 3-5 transfections each out of three independent experiments. Values were normalized to the cleavage rate obtained for the corresponding solvent control (indicated by a gray line). **(C) **Lysates of transfected cells grown in the presence of 0.5 μM of the respective inhibitor were analyzed for Gag processing by quantitative immunoblot using antiserum against HIV CA. Data from one representative replicate are shown.

In order to characterize NNRTI induced PR activation, conditions were optimized for detection of increased, rather than decreased Gag processing. Assuming that the degree of stimulation of Gag-Pol dimer formation is inversely correlated with the intracellular concentration of Gag-Pol [17], β-Gal activity and Gag processing of cells were measured in cells expressing different amounts of HIV derived proteins in the presence or absence of 5 μM EFV as a prototype NNRTI. No effect of EFV was seen at high Gag and Gag-Pol concentrations, whereas transfection of lower amounts of pCHIV.MAα resulted in detectable increase of β-Gal activity in lysates of EFV treated cells (see Additional file 2 for titration data). Under optimized conditions (equal microgram amounts of pCHIV.MAα and pCMVω) enhancement of intracellular Gag processing and a significant increase in β-Gal activity were induced by the addition of 5 μM EFV (Figure 2A, left panels). Cells transfected with a pCHIV.MAα variant in which PR was inactivated due to a D25A mutation in the PR active site (PR-) displayed no increase in Gag processing or β-Gal activity when grown in the presence of 5 μM EFV (Figure 2A, middle panels). As a control mimicking enhanced PR activity we used an HIV-1 derivative expressing an artificially linked PR dimer (2PR). Duplicating the PR monomer coding region in the proviral context and connecting the two PR monomers by a flexible 8 amino acid linker leads to premature activation of HIV PR resulting in greatly enhanced intracellular Gag processing and prevention of virus formation. Low PI doses, which interfere with infectivity of wild-type HIV, partially rescue HIV(2PR) replication by restoring an appropriate level of Gag processing, while high concentrations of PI completely block the activity of the artificially activated PR and lead to the production of non-infectious virus [12,16]. Transfection of a construct encoding the 2PR coding sequence in the context of pCHIV.MAα led to nearly complete intracellular Gag processing (Figure 2A, right panels), while very low levels of CA were released into the supernatant (not shown). No effect of EFV on β-Gal activity was observed in this case, presumably because Gag and Gag-Pol were already completely processed in the absence of EFV (Figure 2A, right panels). Taken together, these results indicate that the EFV mediated increase in β-Gal activity was PR dependent.

In order to identify the most potent available compound we next employed the established assay for a detailed comparison of a series of NNRTIs. We included NNRTIs previously compared qualitatively with respect to activation of Gag processing [27], namely EFV, ETV, NVP and TMC-120 [43], as well as second generation NNRTIs not currently in clinical use: IDX-12899 [44], GW-678248 [45] VRX-480773 [46] and UK-453061 [47]. 293T cells co-transfected with pCHIV.MAα and pCMVω were grown in the presence of the respective NNRTI at concentrations ranging from 0.03 to 10 μM. At 44 h post transfection, cell lysates were analyzed for β-Gal activity. As shown in Figure 2B, compounds varied in their effect: NVP, TMC-120 and UK-453061 displayed little or no enhancement of alpha complementation, while the other compounds tested enhanced β-Gal activity up to 2.5 fold relative to the DMSO control. The most efficient compounds IDX-12899, GW-678248 and VRX-480773 showed strong β-Gal activity enhancement at ~ 250 nM, while ~ 1 μM of ETV or EFV was required to achieve the maximal effect (Figure 2B). At high NNRTI concentrations (5 μM and above) microscopically detectable impairment of cell growth, accompanied by a decrease in β-Gal activity and high signal variability between replicates indicative of cytotoxic effects was observed, and concentrations above 2.5 μM NNRTI were therefore excluded from the analysis shown here; this effect was most pronounced for TMC-120, ETV and VRX-480773. The cytotoxicity observed for TMC-120 under the conditions used, which was confirmed by CC_50 _determination using a T-cell line (see below), likely presents an explanation for a discrepancy between our findings and those of Figueiredo *et al. *[27], who had reported a stimulation of Gag processing upon shorter incubation of cells with 5 μM TMC-120. Under our experimental conditions we could not measure reproducible β-Gal activities at this concentration due to cell death; we can also not exclude that cytotoxicity might have obscured stimulatory effects of TMC-120 at lower concentrations. The ranking in the efficacy of compounds was confirmed by immunoblot analysis of lysates from cells incubated with 0.5 μM of the respective inhibitors (Figure 2C), which showed clear differences between the compounds with respect to the enhancement of Gag processing directly paralleling the results obtained in the alpha complementation assay.

### Selective PR dependent killing of HIV expressing T-cells by NNRTIs

**Figure 3:**
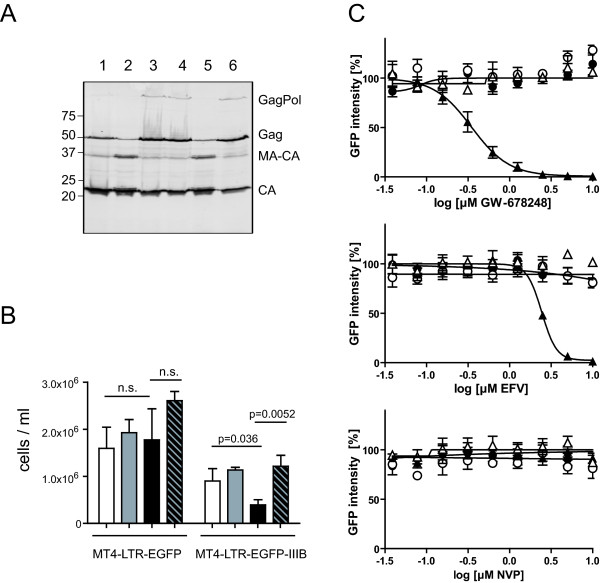
**Intracellular PR activation and NNRTI induced killing of MT-4 cells persistently infected with HIV. (A) **NNRTI induced enhancement of intracellular Gag processing in chronically infected MT-4 cells . MT-4-IIIB cells were cultured in the presence of DMSO (lane 1), 1 μM GW-678248 (lane 2), 200 nM DRV (lane 3), 1 μM GW-678248 + 200 nM DRV (lane 4), 1 μM VRX-480773 (lane 5), or 1 μM VRX-480773 + 200 nM DRV (lane 6), respectively. Cell lysates were harvested and analyzed by immunoblot using antiserum raised against HIV-1 CA. Positions of Gag and Gag-Pol processing products are marked at the right, molecular mass standards are indicated to the left (in kDa). Lysates shown here were harvested at day 2 post addition of compounds; longer incubation periods (6 days) resulted in a more pronounced accumulation of unprocessed Gag in the DRV treated samples, but the pattern in the NNRTI treated samples became difficult to detect due to cell death. **(B) **NNRTI induced killing of chronically infected MT-4 cells. The MT4-LTR-EGFP parental cell line or its persistently HIV-1 infected derivative MT4-LTR-EGFP-IIIB, respectively, were seeded at a density of 1.5 × 10^5 ^cells/ml and incubated for 6 days in the presence of 0.1% DMSO (white bars), 200 nm DRV (gray bars), 1 μM VRX-480773 (black bars) or 1 μM VRX-480773 + 200 nM DRV (hatched bars), respectively. Live cells were counted after trypan blue staining. Data represent mean values and standard deviations from three parallel cultures. P-values were calculated with GraphPad Prism using an unpaired two-tailed t-test. n.s., non significant. **(C) **MT4-CMV-EGFP (circles) or MT4-LTR-EGFP-IIIB (triangles) cells were seeded in 96-well plates at a density of 10^5 ^cells/ml and incubated for 4 days in the presence of various concentrations of the indicated NNRTI, either with (open symbols) or without (filled symbols) the addition of 100 nM DRV. EGFP intensity per well was quantitated at the end of the incubation period by measuring total fluorescence intensity per well based on analysis of microscopic images as described in Methods. The graphs show exemplary data for three NNRTIs. Mean values and standard deviations from three independent wells of one representative experiment are shown. Lines represent fits of the data to a standard dose response equation (4 parameters), yielding CC_50 _values on virus producing cells in the absence of DRV (filled triangles) of 0.35 μM for GW-678248 and 2.44 μM for EFV, respectively. Data from several independent experiments for these compounds as well as for the other NNRTIs were used to calculate the CC_50 _values summarized in Table 1.

The described drug induced PR activation might be exploited to selectively kill HIV infected cells. In order to test this hypothesis, we established the persistently infected T-cell lines MT4-IIIB and MT4-LTR-EGFP-IIIB, where the expression of HIV encoded proteins in >99% of cells could be detected by intracellular p24 staining (not shown). In MT4-LTR-EGFP-IIIB cells, HIV expression could additionally be detected through long terminal repeat (LTR) driven expression of the *gfp *marker gene. As a control we used uninfected MT-4 cells or MT4-CMV-EGFP cells, constitutively expressing EGFP from a CMV promoter, respectively. The use of persistently infected cells enabled us to study the effects of NNRTIs on virus producing cells regardless of their effect on reverse transcription, since the proportion of virus producing cells in this system does not depend on infection of new host cells. Immunoblot analysis of cell lysates after treatment with two of the more potent NNRTIs, VRX-480773 and GW-678248, confirmed that NNRTI mediated enhancement of Gag processing also occurred in virus producing cells, as apparent from the decreased ratio of Gag to intermediate and fully mature processing products (Figure 3A, compare lanes 2 and 5 to lane 1). In order to investigate the effect of NNRTIs on viability of chronically infected cells, MT4-LTR-EGFP-IIIB cells as well as MT4-LTR-EGFP parental cells were treated with 1 μM VRX-480773 for 6 days. Quantification of live cells by microscopic evaluation of trypan blue stained samples revealed a significant decrease in live cell numbers for the HIV infected MT4-LTR-EGFP-IIIB cells, whereas the number of uninfected control cells remained constant (Figure 3B). In order to test whether the observed cytotoxic effect on virus producing cells was due to enhanced HIV PR activity we added 200 nM of the PI darunavir (DRV) to infected and uninfected cells in the presence and absence of VRX-480773. DRV treatment impaired Gag processing (Figure 3A, lanes 3, 4 and 6) and completely reversed the cytotoxic effect of VRX-480773 in MT4-LTR-EGFP-IIIB cells, supporting the interpretation that the observed NNRTI induced cell killing was mediated by HIV PR.

By quantification of intracellular GFP fluorescence of drug treated MT4-CMV-EGFP and MT4-TR-EGFP-IIIB cells, respectively, we compared the relative effect of different NNRTIs on viability of infected versus uninfected cells (Figure 3C and Table 1). Differential effects, correlating with the biochemical data obtained on 293T cells, were revealed (Table 1). The most potent compounds, IDX-12899, GW-678248 and VRX-480773, displayed CC_50 _values in the submicromolar range on MT4-LTR-EGFP-IIIB cells. Cytotoxicity on uninfected MT4-CMV-EGFP control cells was undetectable for IDX-12899 and GW-678248 in the tested range; VRX-480773, displayed detectable unspecific toxicity, albeit with a ~10 fold higher CC_50 _than on virus producing cells. EFV was less cytotoxic on the infected cells, but this effect was again specific as indicated by the observation that MT4-CMV-EGFP cells were not affected. The remaining compounds showed no specific effect in the tested concentration range: TMC-120 displayed toxicity on the virus producing cells, but also showed comparable toxicity on uninfected control cells, while the remaining compounds had no detectable effect on total EGFP expression on either cell line. In all cases the specific NNRTI induced cytotoxicity on virus producing cells was completely reverted by addition of DRV (Table 1).

**Table 1:** Comparison of NNRTI efficacies in various assay systems

	**Inhibition of HIV replication *in vitro *EC**_**50 **_**[nM]**	Enhancement of Gag processing (Fig. 2)	**Cytotoxicity on MT4-CMV-EGFP control cells CC**_**50 **_**[μM]**	**Cytotoxicity on MT4-LTR-EGFP-IIIB HIV-1 producing cells CC**_**50 **_**[μM]**	**Ctotoxicity on MT4-LTR-EGFP-IIIB cells in presence of 0.1 μM DRV CC**_**50 **_**[μM]**	**Enhancement of RT-Dimerization EC**_**50 **_**[μM]**
IDX-12899	1.9 ± 1.3	++	> 10	0.29 ± 0.21	> 10	0.0046

GW-678248	0.84 ± 0.25	++	> 10	0.63 ± 0.29	> 10	0.0032

VRX-480773	1.6 ± 0.81	++	5.82 ± 1.44	0.68 ± 0.34	6.33 ± 0.08	0.0040

EFV	1.9 ± 0.9	+	> 10	1.71 ± 0.43	> 10	0.020

ETV	3.2 ± 5	+	> 10	> 10	> 10	0.27

UK-453061	7.5 ± 1.4	-	> 10	> 10	> 10	0.15

NVP	42 ± 20	-	> 10	> 10	>10	18

TMC-120	1.7 ± 1.4	-	3.02 ± 0.90	2.56 ± 0.74	4.33 ± 0.81	ND

*mean values and standard deviations from three or more independent measurements are shown; ND, not done.

These results support the hypothesis that NNRTIs can exert a dose dependent, inhibitor specific activation of intracellular HIV PR by stabilizing Gag-Pol dimers. In order to obtain further evidence for this model, we analyzed the effect of the various NNRTIs on RT dimerization in a mammalian two-hybrid system [48]. We found that, while lower absolute concentrations were required in this context, the relative effects of the various compounds on RT dimer formation paralleled their effects on intracellular Gag processing: IDX-12899, GW-678248 and VRX-480773 promoted RT dimerization in the low nM range, whereas a fivefold higher concentration was required for EFV, and EC_50 _values for the remaining compounds were higher than 100 nM (Table 1; see Additional file 3 for exemplary primary data). This correlation lends further support to the proposed mechanism of action.

**Figure 4:**
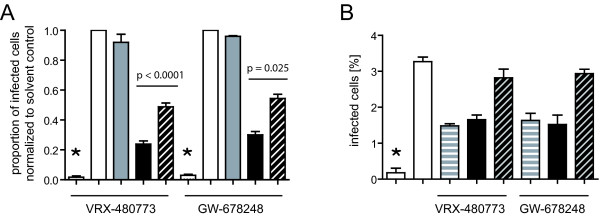
**NNRTI induced selective killing of HIV-1 infected primary human cells**. **(A) **PBMC prepared from buffy coats of healthy blood donors were infected with HIV-1AGFP. At day 2 post infection, 100 ng/ml AMD-3100 was added to all samples to prevent further infection. Individual samples were incubated in addition with DMSO (white bars), 200 nm DRV (gray bars), 1 μM of the indicated NNRTI (black bars) or 1 μM NNRTI + 200 nM DRV (hatched bars), respectively. After further incubation for 5 days, cells were harvested and analyzed for the proportion of infected GFP expressing cells by flow cytometry. The figure shows mean values and standard deviations from three independent experiments (VRX-480773) or one experiment (GW-678248), respectively, each comprising three parallel cultures using different donor pools. P-values were calculated using a two-tailed unpaired t-test (GraphPad Prism). Values were normalized to the respective solvent control. **(B) **CD4 positive cells isolated from PBMC were infected with HXB2D-EGFP. At day 7 post infection 1 μM AZT (white bars), 1 μM of the indicated NNRTI (striped bars), 1 μM of the indicated NNRTI + 1 μM AZT (black bars) or 1 μM NNRTI + 1 μM AZT + 100 nM DRV (hatched bars), respectively, were added. After further incubation for 3 days, cells were harvested and analyzed for the proportion of infected cells by flow cytometry. The figure shows mean values and standard deviations of values from one representative experiment (three parallel infections). Asterisks: non-infected controls.

To validate our results obtained for the persistently infected cell line in a more relevant cell system we performed additional infection experiments using human peripheral blood mononuclear cells (PBMC). In these experiments we focused on two of the most potent compounds, GW-678248 and VRX-480773, which displayed CC_50 _values in the sub-micromolar range on virus producing MT-4 cells (Table 1). PBMC isolated from healthy blood donors were activated and infected with a replication competent HIV-1 derivative which carries a *gfp *gene in the *nef *locus [49]. The co-receptor antagonist AMD-3100 was added at day 2 post infection to prevent further viral spread. This was done to distinguish the proposed killing of infected cells from the inhibitory effect of NNRTIs and PIs on virus replication. At the time of AMD-3100 addition, individual samples were further treated with solvent only, 1 μM NNRTI, 200 nM DRV, or a mixture of both. The percentage of infected cells was determined following incubation for 5 days by flow cytometry (Figure 4A) yielding values between 2 and 6% for the control samples. Analogous to our results with the MT-4 cell line (compare Figure 3B) we observed a significant reduction of infected primary cells upon treatment with VRX-480773 or GW-678248 as compared with the control. This effect was partially reversed by addition of PI and thus dependent on PR activity (Figure 4A). Rescue was incomplete, however, despite a complete blockage of Gag processing by DRV under these conditions (see Additional file 4 for immunoblot analysis). Similar results were obtained upon infection of CD4-positive primary T-cells with an EGFP-expressing virus (Figure 4B). In this case, AZT was used to prevent ongoing viral spread, but the same PR dependent cytotoxicity was observed upon addition of either 1 μM GW-678248 or 1 μM VRX-480773. In this case, the addition of DRV completely reversed the NNRTI effect, indicating that the induced cytotoxicity was largely dependent on PR activity.

## Discussion

Triggered by previous reports that certain NNRTIs can enhance HIV-1 PR activity, the present study provides proof of principle that this effect can be exploited for the specific killing of HIV producing cells in tissue culture. Applying a newly developed enzymatic assay measuring intracellular HIV PR activation we compared relative activities of various NNRTIs on intracellular Gag and Gag-Pol processing. These activities correlated with the potency of the respective compounds to enhance intracellular RT heterodimerization and, more importantly, with their efficacy regarding specific killing of HIV producing cells. Similar effects were obtained for chronically HIV-1 infected MT-4 cells and for acutely infected PBMC, indicating that the observed effects are not cell-type dependent and may occur at different levels of HIV-1 gene expression.

Efficient intracellular PR activation is apparently not a general property of NNRTIs. The relative efficacies varied and three NNRTIs tested did not display detectable effects under the conditions used here. The structural basis for these differences in PR activating potential between the various NNRTIs is currently not clear. The fact that this potential did not correlate with the relative antiviral efficacies of the respective compounds at lower concentrations mediated by inhibition of RT enzymatic activity suggests that the two activities are structurally distinct. This may be related to the relative affinities of the compounds to mono- or dimeric forms of the enzyme [32] and these features may be exploited for the development of derivatives with increased activity.

Anti-infective drugs acting not, or not exclusively, on viral replication, but rather affecting virus producing cells may be considered for strategies aimed at HIV eradication from the infected organism. Despite efficient long term suppression of HIV by current therapies, virus eradication is not achieved, most likely because of reservoirs of long-lived latently infected cells [50-52]. HIV gene expression is an obvious requirement for the NNRTI enhanced PR cytotoxicity described in the current study, and transcriptionally silent cells harbouring HIV proviral DNA can thus not be directly targeted. This approach may be synergistic, however, with the proposed activation of latent reservoirs by small molecules (e.g. affecting chromatin structure). The activation should induce HIV expression in the absence of global T-cell activation, while the spread of infection to new target cells is prevented by available antiretroviral drugs [53]. A combination of this strategy with targeted PR activation would of course require the use of PI sparing HAART regimens [54] for prevention of viral spread; a regimen lacking PI and containing NNRTIs with a high potential for PR activation may be optimal to exploit the observed cytotoxic activity in such a situation. Induced killing of HIV-1 infected cells may also be exploited to target persistent reservoirs of HIV producing cells. The existence of such reservoirs that differ from latently infected cells is suggested by the continuous presence of very low viral loads under therapy, which do not respond to HAART treatment intensification [3,55,56]. While the nature of these reservoirs is uncertain, a strategy for targeted PR activation may contribute to diminish or eliminate these virus producing cells.

Previous studies had reported EFV to be the most efficient NNRTI with respect to PR activation. Although we were able to identify inhibitors in clinical development displaying a higher efficacy than EFV and showed that these higher efficacies translated into a detectable specific cytotoxicity on HIV producing cells in tissue culture, CC_50 _values determined were still in the high nanomolar range. Peak serum levels of EFV are in the micromolar range [57], suggesting that the proposed mechanism of NNRTI induced killing of HIV-1 producing T-cells might already occur *in vivo *under therapy. Nevertheless, the therapeutic window between specific and unspecific cytotoxicity is likely to be rather narrow for most NNRTIs and thus more potent compounds will be required for development of this inhibitory mechanism into an applicable therapeutic strategy. A peptide (P_AW_) which stabilizes RT dimers and displays potent antiviral activity *in vitro *has also been described [58]. Since P_AW _appears to interact with a site not overlapping the NNRTI binding pocket, it points to another potential target site for enhancers of Gag-Pol dimer stabilization. However, P_AW _has so far only been reported to interact with the dimeric forms of RT; it remains to be investigated whether this peptide - or compounds targeting the same binding site on RT - could also promote Gag-Pol dimer formation.

## Conclusion

In summary, the results presented here are consistent with the following model, which we propose as a working hypothesis as a basis for further investigation: certain NNRTIs can increase intracellular Gag-Pol dimer concentration upon binding to the RT domain of Gag-Pol and thereby stimulate intracellular PR activity. Enhanced activation of PR reduces virion formation through depletion of the assembly competent Gag and Gag-Pol precursor proteins, as shown in earlier studies [12,16,17,27], but furthermore leads to the death of the virus expressing cell, as presented in this study. Based on the proposed mechanism, a small molecule compound which efficiently enhances Gag-Pol dimerization would have a dual and synergistic effect on HIV spread in directly preventing virus production on one side and accelerating the death of virus producing cells on the other. The data presented here provide proof of concept for a drug induced killing of HIV producing cells, but more potent inducers of Gag-Pol dimerization will likely be required for therapeutic application, especially for targeting cells expressing low amounts of Gag-Pol. The current incomplete knowledge of the Gag-Pol dimerization process and of other mechanisms involved in PR activation prevents a rational search for PR activating compounds; however, the gel independent assay described here may provide a basis for screening of compound libraries for such activities. Alpha complementation has successfully been used in various high throughput screening approaches [39] and it appears likely that more potent enhancers of Gag-Pol dimerization and PR activation can be identified based on this method. Such novel compounds may ultimately render selective killing of HIV-1 infected cells by increased PR toxicity a feasible therapeutic approach.

## Methods

### Plasmids

HIV-1 proviral constructs were based on plasmid pNLC4-3 [59] and non-infectious virus variants were derived from the previously described plasmid pCHIV, a CMV promoter driven derivative of NL4-3 lacking both HIV LTR regions [41]. The coding sequence for amino acids 1-51 of β-Gal from *Escherichia coli*, amplified by PCR from plasmid pCMVbeta (Invitrogen) and flanked at the N-terminus by a coding sequence for a HIV-1 PR recognition site, was cloned into engineered unique *BspEI *and *AfeI *restriction sites which had been inserted into pCHIV between codons 128 and 129 of MA (see Figure 1A for resulting amino acid sequences). The 2PR derivatives of pCHIV and pCHIV.MAα were cloned by exchange of an ApaI fragment against the respective fragment from plasmid pNL4-3.2PR [16]. Plasmid pCMVω was constructed by amplifying the β-Gal encoding sequence from plasmid pCMVbeta by PCR, using an N-terminal primer that introduced a deletion of codons 11-41 (primer sequence: GGCGCCATGGGCGTGATCACCGACAGCCTGGCCGTGGAGGCCCGCACCGATCGCCC). The resulting ω-fragment encoding PCR fragment was cloned into the *EcoRV *site of pcDNA3.1Zeo by blunt end ligation. Expression of a protein of the expected molecular mass was confirmed by immunoblot using polyclonal antiserum against β-Gal (Abcam ab 616; not shown).

### Cells and viruses

MT4-CMV-EGFP and MT4-LTR-EGFP cells were obtained by transfection of MT-4 cells with a selectable construct comprising the *egfp *gene under the control of a CMV promoter or the HIV-1 long terminal repeat (LTR) region, respectively, and subsequent selection of stably transfected cells. Persistently infected MT4-IIIB and MT4-LTR-EGFP-IIIB cells were generated by infection of parental MT-4 or MT4-LTR-EGFP cells, respectively, with HIV-1IIIB at an MOI of 0.1. The cytopathic effect of HIV led to a dramatic cell loss early after infection, but persistently infected MT4-IIIB and MT4-LTR-EGFP-IIIB cells, displaying a similar morphology as the parental cells and only slightly delayed proliferation could be selected within 2-3 weeks post infection. Persistent productive infection with HIV-1 was demonstrated by the detection of infectious virus in the tissue culture supernatant and intracellular anti-p24 staining, as well as by syncytia formation upon mixing with non-infected MT-4 cells. All MT-4 derived cell lines as well as C8166 cells were maintained in RPMI 1640 medium supplemented with 10% heat-inactivated fetal calf serum, 2 mM L-glutamine, 0.1% NaHCO3, and 0.02% gentamycin.

Peripheral blood mononuclear cells (PBMC) were purified from buffy coats of HIV-negative blood donors, grown in supplemented RPMI 1640 and stimulated by the addition of 10 ng/ml IL-2 (Biomol) and 2 μg/ml PHA (Sigma). PBMC pooled from two donors each were used for infection. CD4 positive cells from the PBMC pool activated as previously described (*Division of AIDS, National Institute of Allergy and Infectious Diseases, National Institutes of Health, and Collaborating Investigators. 1997. Virology manual for HIV laboratories. Publication NIH-97-3828. U.S. Department of Health and Human Services,Washington D.C.*) were isolated by magnetic sorting using anti-CD4 magnetic microbeads (Miltenyi Biotec) according to the manufacturer's instructions. For infection of PBMC, the HIV-1 derivatives HIV-1-AGFP [49] carrying the *gfp *gene fused to the codon for amino acid 16 of Nef in pNL4-3, or HXB2D-EGFP [60], which carries an *egfp *gene in the place of the viral *nef *open reading frame, were used as indicated. Virus stocks were prepared by transfection of the respective proviral plasmids in 293T cells.

### Inhibitors

EFV, LPV, DRV, ETV, NVP and AMD-3100 were obtained through the AIDS Research and Reference Reagent Program, Division of AIDS, NIAID, NIH. IDX-12899 [44], GW-678248 [45], VRX-480773 [46], UK-453061 [47] and TMC-120 [43] were synthesized at Tibotec. Compounds were dissolved and stored as 10 mM stock solutions in 100% DMSO and diluted with tissue culture medium to the final concentration immediately before use.

### Analysis of Gag expression, processing and particle release

293T cells were seeded in 6-well plates and transfected with the indicated constructs using FuGene6 (Roche) according to the manufacturer's instructions. Cell lysates and tissue culture supernatants were harvested at 44-48 h post transfection. Virus was purified by ultracentrifugation through a 20% (w/w) sucrose cushion. Cell lysates, tissue culture supernatants or pelleted viral particles were separated by SDS-PAGE (17.5% acrylamide; acrylamide:bisacrylamide 200:1). Proteins were transferred to nitrocellulose by semi-dry blotting and detected using polyclonal antisera raised against recombinant HIV-1 CA or MA, or a commercial antiserum against β-Gal (Abcam, ab616), respectively. Detection of bound antibody by quantitative immunoblot was carried out with a LiCor Odyssey system using protocols and secondary antibodies suggested by the manufacturer and evaluated using Odyssey v2.0 detection software.

### Measurement of β-Gal activity in cell lysates

The activity of β-Gal in cell lysates from transfected 293T cells was measured by enzymatic cleavage of the chromogenic β-Gal substrate chlorphenolred-β-D-galactopyranoside (CPRG, Roche; [42]). At 44 h post transfection, cells were briefly rinsed with PBS and suspended in reporter gene assay lysis buffer (Roche, 600 μl per 6-well dish) supplemented with a protease inhibitor mix (Roche). Cell suspensions were incubated for 10 min at room temperature and cell debris was subsequently removed by brief centrifugation. Five μl of supernatant were diluted in 96-well plates with 95 μl CPRG reaction buffer (50 mM potassium phosphate, pH 7.5, 1 mM MgCl_2_) and pre-warmed for 5 min to 37°C. 100 μl of pre-warmed reaction mix (100 μM CPRG in CPRG reaction buffer supplemented with protease inhibitor cocktail and 40 μM β-mercaptoethanol) were added and β-Gal mediated cleavage of CPRG was monitored by recording absorption at 592 nm every 2 min for 20 min at 37°C using a TECAN Safire multi-well reader. OD592 values were plotted over time and relative reaction rates (OD592/min) were determined from the initial linear velocities.

### Determination of direct antiviral activity and cytotoxicity

MT4-LTR-EGFP cells were seeded at a density of 1.5 × 10^5 ^cells/ml and infected with HIV-1IIIB at a multiplicity of infection of 0.01 in the presence of different NNRTI concentrations. After 3 days of incubation, infected cells were quantified by determination of total EGFP fluorescence per well based on microscopy and subsequent image analysis. Threshold values were determined from the average pixel value plus 6 standard deviations from the uninfected control wells, and the median threshold from all control wells on a plate was defined as baseline GFP expression. Intensity values for the sample wells were then determined by subtracting the background threshold from each pixel value obtained from the image of the respective well and calculating the sum of net pixel intensities. Percent inhibition was calculated as 100 * (1 - (Sample - CC)/(VC - CC)). The 50% effective concentration (EC_50_) was calculated by fitting the data to a standard dose response equation and is defined as the concentration that reduced virus induced fluorescence by 50% as compared to the DMSO control. Data shown in Table 1 represent mean values of at least three independent experiments.

The cytotoxicity of inhibitors was determined in parallel on MT4-CMV-EGFP control cells and on MT4-LTR-EGFP-IIIB virus producing cells, respectively. Cells were seeded into 96-well plates at a density of 1.5 × 10^5 ^cells/ml and grown for 4 days in the presence or absence of different compound concentrations. Cell proliferation was quantified by measuring the EGFP fluorescence per well based on microscopy followed by image analysis as described above and expressed as CC_50 _values calculated by fitting the data to a standard dose response equation (drug concentration which led to reduction of cell associated fluorescence by 50%).

### Determination of enhancement of RT dimerization

RT heterodimer formation was monitored using a mammalian two hybrid system described previously [48]. In brief, the bait protein (p66) was fused to the C-terminus of a chimeric receptor consisting of the extracellular part of the erythropoietin receptor and the intracellular part of the leptin receptor incapable of STAT activation. The prey protein (p51) was coupled to a part of the cytoplasmic tail of the gp130 chain carrying several STAT3 recruitment domains. Interaction of bait and prey protein leads to functional complementation of STAT3 activity, which results in Epo dependent induction of a STAT3-responsive luciferase reporter gene. Enhancement of this interaction by the addition of compounds can thus be measured by an increase of luciferase expression. The compound concentration which resulted in enhancement of the signal by 50% was reported as EC_50 _in Table 1.

## Competing interests

This study was supported by Tibotec Pharmaceuticals, Ltd. DJ, IK, LS and GK are employed by Tibotec-Virco BVBA, Mechelen, Belgium.

## Authors' contributions

DJ and BM performed initial experiments and cooperated in study design and coordination. MA carried out alpha complementation assays and experiments on specific cytotoxicity in infected MT-4 cells and PBMC. IK and LS performed experiments on sorted PBMC and comparison of the complete NNRTI panel with respect to cytotoxicity levels on MT-4 cells and RT dimerization. GK and HGK participated in study design, discussion and coordination. BM drafted the manuscript with help of DJ and HGK. All authors read and approved the final manuscript.

## Supplementary Material

Additional file 1 **Infectivity of HIV.MAα (A) **HIV_NL4-3 _and HIV_NL4-3_MAα harvested from transfected 293T cells were used to infect C8166 cells. At days 3 to 7 post infection, samples from the tissue culture supernatant were harvested and the amount of p24 CA was determined by quantitative immunoblot. The graph shows mean values and standard deviations from three independent infections from one representative experiment (wild-type HIV, filled triangles; HIV.MAα , open triangles; mock infected cells, open circles), respectively. **(B) **Integrity of the reporter virus after several rounds of replication was verified by immunoblot of lysate from infected cells. At day 7 post infection, cell lysates from the infection experiment shown in (A) were harvested and analyzed by immunoblot using the indicated antisera. The presence of the slower migrating form of MA carrying the linker sequence (MA*) as well as of a slightly slower migrating form of Gag (Gag.MAα) indicates that the peptide insertion was retained. Click here for file

Additional file 2 **Effect of EFV on Gag processing (A) and β-Gal activity (B) in cell lysates **293T cells were seeded in 6-well plates, transfected with the indicated ratio of pCHIV.MAα and pCMVω and incubated in the absence (-, white bars) or presence (+, black bars) of 5 μM EFV, respectively. **(A) **At 44 h post transfection, cell lysates and virus particles pelleted from the supernatant by ultracentrifugation were harvested and analyzed by immonoblot using antiserum raised against HIV-1 CA. Data from one representative experiment are shown. **(B) **In parallel, samples of cell lysates were analyzed for β-Gal activity as described in methods. The graph shows mean values and standard deviations from three independent transfections from one representative experiment. Click here for file

Additional file 3 **Enhancement of RT heterodimer formation by NNRTIs **RT heterodimer formation in cells treated with different concentrations of NNRTIs was assayed using a mammalian two-hybrid system (MAPPIT, [48]) as described in Methods. Enhancement of luciferase reporter gene activities relative to the DMSO control was plotted and used to calculate EC_50 _values, defined as an enhancement of 50% over the control value. The graph shows representative dat sets for titrations with NVP (diamonds), EFV (triangles) and VRX-480773 (circles), respectively. Several independent experiments for each NNRTI tested were performed to calculate CC_50 _values summarized in Table 1. Click here for file

Additional file 4 **Efficacy of PR inhibitor treatment on infected PBMC **Representative samples from the experiment shown in Figure 4 were analyzed by immunoblot of cell lysates harvested at the end of the experiment using antiserum raised against HIV-1 CA. The figure shows samples of unifected cells (lane 1), as well as infected cells treated with AMD-3100 (lane 2), AMD-3100 + DRV (lane 3), AMD-3100 + VRX-480773 (lane 4) and AMD-3100 + VRX-480773 + DRV (lane 5), respectively. Samples corresponding to equal tissue culture volumes were loaded. Click here for file
