# Supplementary figures and images for: Selective killing of human immunodeficiency virus infected cells by non-nucleoside reverse transcriptase inhibitor-induced activation of HIV protease
Source: Retrovirology. 2010 Oct 15;7:89. doi: 10.1186/1742-4690-7-89 (PMC2974656; doi:10.1186/1742-4690-7-89)

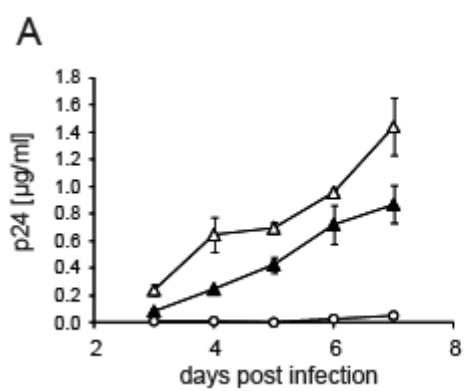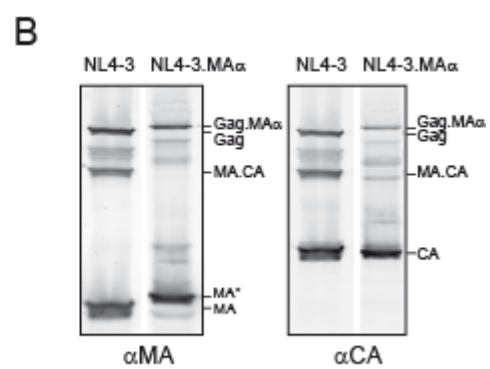

Supplement: Additional file 1 — Infectivity of HIV.MAα (A) HIVNL4-3 and HIVNL4-3MAα harvested from transfected 293T cells were used to infect C8166 cells. At days 3 to 7 post infection, samples from the tissue culture supernatant were harvested and the amount of p24 CA was determined by quantitative immunoblot. The graph shows mean values and standard deviations from three independent infections from one representative experiment (wild-type HIV, filled triangles; HIV.MAα , open triangles; mock infected cells, open circles), respectively. (B) Integrity of the reporter virus after several rounds of replication was verified by immunoblot of lysate from infected cells. At day 7 post infection, cell lysates from the infection experiment shown in (A) were harvested and analyzed by immunoblot using the indicated antisera. The presence of the slower migrating form of MA carrying the linker sequence (MA*) as well as of a slightly slower migrating form of Gag (Gag.MAα) indicates that the peptide insertion was retained. [file 1742-4690-7-89-S1.pdf]

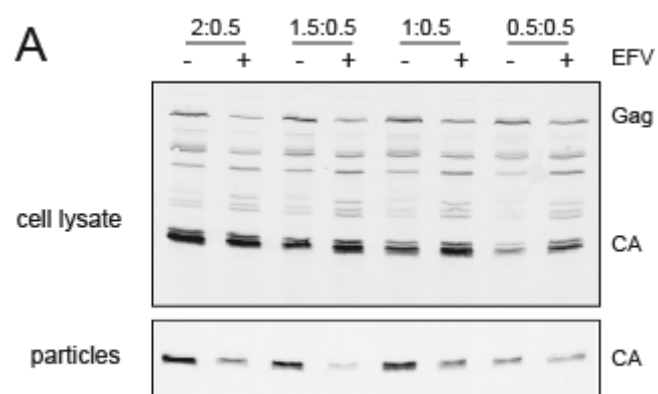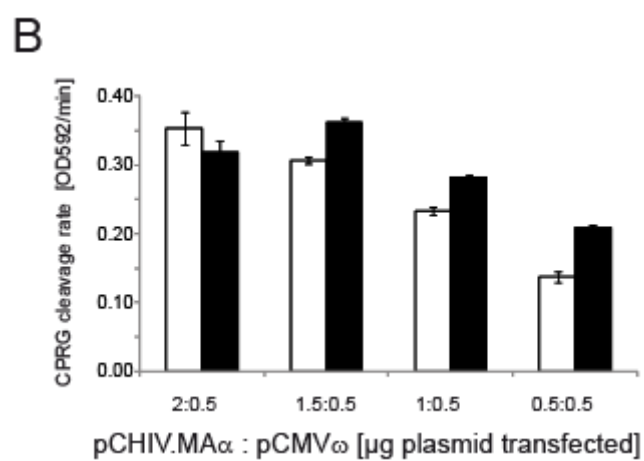

Supplement: Additional file 2 — Effect of EFV on Gag processing (A) and β-Gal activity (B) in cell lysates 293T cells were seeded in 6-well plates, transfected with the indicated ratio of pCHIV.MAα and pCMVω and incubated in the absence (-, white bars) or presence (+, black bars) of 5 μM EFV, respectively. (A) At 44 h post transfection, cell lysates and virus particles pelleted from the supernatant by ultracentrifugation were harvested and analyzed by immonoblot using antiserum raised against HIV-1 CA. Data from one representative experiment are shown. (B) In parallel, samples of cell lysates were analyzed for β-Gal activity as described in methods. The graph shows mean values and standard deviations from three independent transfections from one representative experiment. [file 1742-4690-7-89-S2.pdf]

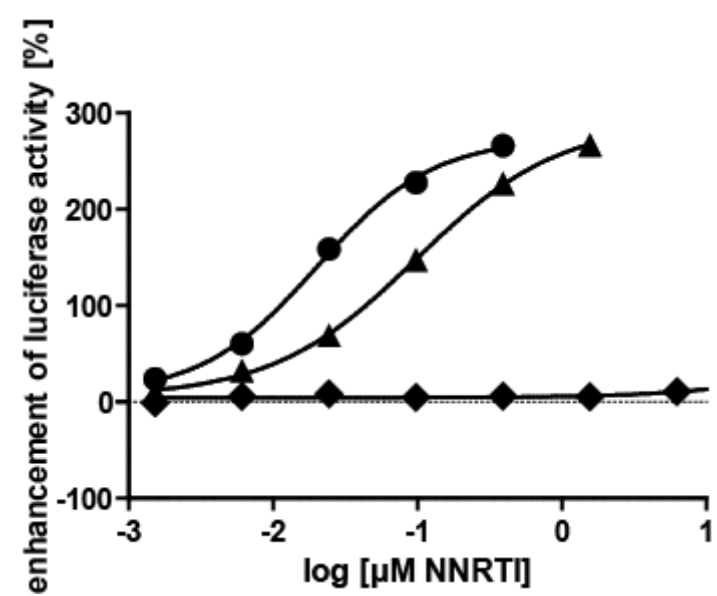

Supplement: Additional file 3 — Enhancement of RT heterodimer formation by NNRTIs RT heterodimer formation in cells treated with different concentrations of NNRTIs was assayed using a mammalian two-hybrid system (MAPPIT, [48]) as described in Methods. Enhancement of luciferase reporter gene activities relative to the DMSO control was plotted and used to calculate EC50 values, defined as an enhancement of 50% over the control value. The graph shows representative dat sets for titrations with NVP (diamonds), EFV (triangles) and VRX-480773 (circles), respectively. Several independent experiments for each NNRTI tested were performed to calculate CC50 values summarized in Table 1. [file 1742-4690-7-89-S3.pdf]

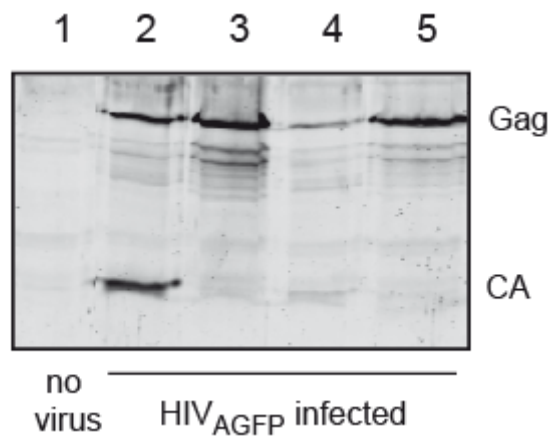

Supplement: Additional file 4 — Efficacy of PR inhibitor treatment on infected PBMC Representative samples from the experiment shown in Figure 4 were analyzed by immunoblot of cell lysates harvested at the end of the experiment using antiserum raised against HIV-1 CA. The figure shows samples of unifected cells (lane 1), as well as infected cells treated with AMD-3100 (lane 2), AMD-3100 + DRV (lane 3), AMD-3100 + VRX-480773 (lane 4) and AMD-3100 + VRX-480773 + DRV (lane 5), respectively. Samples corresponding to equal tissue culture volumes were loaded. [file 1742-4690-7-89-S4.pdf]
